# Specific age‐correlated activation of top hierarchical motor control areas during gait‐like plantar stimulation: An fMRI study

**DOI:** 10.1002/hbm.25691

**Published:** 2021-11-05

**Authors:** Henry Jeanvoine, Matthieu Labriffe, Thomas Tannou, Nastassia Navasiolava, Aram Ter Minassian, Jean‐Baptiste Girot, Louis‐Marie Leiber, Marc‐Antoine Custaud, Cédric Annweiler, Mickaël Dinomais

**Affiliations:** ^1^ Department of Radiology, Angers University Hospital University of Angers Angers France; ^2^ Laboratoire Angevin de Recherche en Ingénierie des Systèmes, EA7315 University of Angers Angers France; ^3^ Department of Geriatrics, Besançon University Hospital University of Franche‐Comté Besançon France; ^4^ Integrative and Clinical Neurosciences, EA 481 University of Franche‐Comté Besançon France; ^5^ Centre de Recherche Institut Universitaire de Gériatrie de Montréal Montréal Canada; ^6^ Clinical Research Center, Angers University Hospital University of Angers Angers France; ^7^ Department of Anesthesia and Critical Care Angers University Hospital Angers France; ^8^ Laboratoire de Biologie Neuro‐Vasculaire et Mitochondriale Intégrée, UMR CNRS 6214 INSERM U1083 University of Angers Angers France; ^9^ Department of Neuroscience, Division of Geriatric Medicine and Memory Clinic—Angers University Hospital UPRES EA 4638—University of Angers Angers France; ^10^ Department of Medical Biophysics, Robarts Research Institute, Schulich School of Medicine and Dentistry University of Western Ontario London Ontario Canada; ^11^ Department of Physical and Rehabilitation Medicine, Angers University Hospital University of Angers Angers France

**Keywords:** aging, brain, functional magnetic resonance imaging, functional neuroimaging, gait, motor cortex

## Abstract

A better understanding of gait disorders that are associated with aging is crucial to prevent adverse outcomes. The functional study of gait remains a thorny issue due to technical constraints inherent to neuroimaging procedures, as most of them require to stay supine and motionless. Using an MRI‐compatible system of boots reproducing gait‐like plantar stimulation, we investigated the correlation between age and brain fMRI activation during simulated gait in healthy adults. Sixty‐seven right‐handed healthy volunteers aged between 20 and 77 years old (49.2 ± 18.0 years; 35 women) were recruited. Two paradigms were assessed consecutively: (a) gait‐like plantar stimulation and (b) chaotic and not gait‐related plantar stimulation. Resulting statistical parametric maps were analyzed with a multiple‐factor regression that included age and a threshold determined by Monte‐Carlo simulation to fulfill a family‐wise error rate correction of *p* < .05. In the first paradigm, there was an age‐correlated activation of the right pallidum, thalamus and putamen. The second paradigm showed an age‐correlated deactivation of both primary visual areas (V1). The subtraction between results of the first and second paradigms showed age‐correlated activation of the right presupplementary motor area (Brodmann Area [BA] 6) and right mid‐dorsolateral prefrontal cortex (BA9‐10). Our results show age‐correlated activity in areas that have been associated with the control of gait, highlighting the relevance of this simulation model for functional gait study. The specific progressive activation of top hierarchical control areas in simulated gait and advancing age corroborate a progressive loss of automation in healthy older adults.

## Graphical abstract

Gait decline during normal aging is a key public health concern. This fMRI study addresses the question of brain activation evolution with advance in age in healthy adults. We highlight a gradual and specific overactivation of top hierarchical cortical motor control areas in simulated gait during advance in age. Our results support the theory of a progressive loss of automation of gait in older healthy adults.

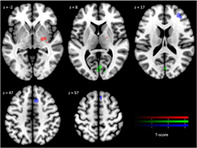

## 1 INTRODUCTION

Gait decline is commonly described during normal aging. Though aging is accompanied by a richer knowledge reserve and skillset based on a multitude of life experiences, these gains are offset by changes in sensory, motor, and cognitive processing due to alterations in the functioning of the central nervous system as well as other physiological systems (Harada, Natelson Love, & Triebel, 2013; Paraskevoudi, Balcı, & Vatakis, 2018). Excess gait decline over time is independently associated with early detection of physical frailty and its many adverse outcomes, among which increased risks of falls, hospitalization, disability, morbidity, and mortality (Newman et al., 2006; Verghese, Holtzer, Lipton, & Wang, 2009) but also cognitive impairment and dementia (Grande et al., 2019). Therefore, gait control goes beyond a motor issue to become part of a complex cognitive process. In this respect, age‐related gait changes could be used as early markers of cognitive decline and loss of autonomy (Åkerborg et al., 2016; Axer, Axer, Sauer, Witte, & Hagemann, 2010; Heinrich, Rapp, Rissmann, Becker, & König, 2010).

Gait is an integrated process consisting of rhythmic movements constantly adapted to the environment by the continuous integration of multimodal sensory information (Rossignol, Dubuc, & Gossard, 2006). It is controlled by a hierarchical network of locomotion centers. At the lowest level, gait involves key circuits known as spinal locomotor “central pattern generators” (CPGs) generating and fine‐tuning limb movements through the local integration of proprioceptive and somesthetic inputs (Grillner, 2006). At the supraspinal level, these CPGs are controlled by hierarchically organized locomotion centers located in the brainstem, cerebellum, thalamus, and basal ganglia, which can initiate, scale, sustain, and stop the descending command for walking (Le Ray, Juvin, Ryczko, & Dubuc, 2011). At the highest level, the brainstem, thalamus, and basal ganglia centers relay information to the cortex, and their activity is modulated by cortical structures, for example, sensory (visual, auditory, vestibular) areas, temporal areas for spatial orientation, or pre‐frontal areas for supervision (Rossignol et al., 2006).Finally, the primary motor cortex (M1) plays an essential role in facilitating movements (Yang & Gorassini, 2006). However, recent evidence (McCrimmon et al., 2018) demonstrated that the cognitive control of gait is not only complex, but that it also involves a variety of neural networks.

Although gait processes appear to be automated, this automation involves a complex network of neural structures. Automaticity represents the ability of the nervous system to successfully coordinate movements with minimal use of attention‐demanding executive control resources (Clark, 2015). While automatic processing requires little effort and can operate in high workload situations, controlled/executive processing requires substantial effort and interferes with other controlled processing tasks (Schneider & Chein, 2003). Gait automaticity is seen as a hallmark of a healthy control of walking (Clark, 2015), with prefrontal cortex proposed as a region of main interest, operating at the highest levels of the control hierarchy for cognitive and motor functions (Koechlin, Ody, & Kouneiher, 2003).

Whereas the description of the neural structures involved in gait, as mentioned above, is now relatively consensual, it remains unclear how aging of these neural structures explains gait decline (Wilson, Allcock, Mc Ardle, Taylor, & Rochester, 2019). As reviewed by Holtzer, Epstein, Mahoney, Izzetoglu, and Blumen (2014), neuroimaging studies of gait in aging remain scarce, heterogeneous, and involve diverse paradigms, such as real or imagined walking tasks (la Fougère et al., 2010) of various complexity, like dual‐tasks (Blumen, Holtzer, Brown, Gazes, & Verghese, 2014), backwards walking (Godde & Voelcker‐Rehage, 2010), and obstacle avoidance (Wai et al., 2012). These experimental tasks were performed by healthy or demented adults, and several functional neuroimaging techniques were used including functional magnetic resonance imaging (fMRI), near infra‐red spectroscopy (fNIRS), and positron emission tomography (PET). The compilation of these studies shows some general tendencies between cognitively healthy younger and older adults, mainly a wider‐spread cortical control of locomotion in older adults with an activation of frontal cortical regions in imagined walking and increased task complexity (Holtzer et al., 2014). As such, two regions of interest seem particularly involved in gait management in older adults, that is, the right supplementary motor area (SMA, BA6), also known as pre‐SMA, and the mid‐dorsolateral frontal precortex (mid‐DLFPC) (Holtzer et al., 2014). This is in line with the hypothesis of a gait decline by automaticity loss in older adults, though more consistent results are needed to support this theory.

Despite substantial capabilities, functional neuroimaging techniques each have drawbacks. For example, fNIRS provides continuous, noninvasive, unobtrusive monitoring that can be used in real walking, but is limited to superficial recording of cortex and has low spatial resolution compared to fMRI (Clark, 2015). In comparison, fMRI is a tool of choice as a noninvasive, tridimensional, and high‐resolution technique. Though, gait study in fMRI is challenging by requiring the participant to be immobile in a supine position during gait assessment, which is incompatible with real gait assessment (Labriffe et al., 2017). Therefore, a variety of protocols have been developed involving various gait‐related tasks like motor (Jeannerod, 1995; Porro et al., 1996), visual, and kinetic imagery (Solodkin, Hlustik, Chen, & Small, 2004), with the aim to be as functionally similar as possible to execution. These imagery tasks necessarily involve (a) heterogeneity in simulated gait paradigms and (b) significant differences between simulated and real gait activation pattern. However, the paradigms used in fMRI to date have been mainly based on the visual or cognitive projection of walking activity. As such, even if the correlation between these paradigms and actual walking is well recognized, the investigation of gait decline remains very limited in these settings. In particular, while the research hypotheses described above are in favor of an alteration in gait automaticity during aging, current tools limit the capacity for an in‐depth exploration of these elements.

Recently, to go beyond these limits, we proposed a novel protocol using an MRI‐compatible system of boots, the Korvit boot system (Kremneva et al., 2012), inducing passive mechanical stimulation of the plantar surface of the foot which reproduces gait‐like plantar stimulation, fully reproducible among subjects. Even if it was not directly designed to study gait cognitive control, but to study the brain activations related to a simulated gait, it turns out that, in a previous paper, it has been demonstrated that the Korvit system could mimic sensations associated with walking since it reproduced activations compatible with real and simulated gait (Labriffe et al., 2017). Indeed, in this previous manuscript, it was shown that brain activations related to the somatosensory integration of a simulated step sequence activated the cognitive areas of walking, in particular the primary sensorimotor cortex and the secondary somatosensory cortex bilaterally, but also the bilateral SMA‐proper and the right pre‐SMA, underlining the potential key role of the SMA in gait control.

To date, no large‐scale study has been designed to address changes in brain activation associated with aging in healthy adults by using gait‐like peripheral stimulation setting. In order to contribute to the exploration of the neurofunctional substrates associated with gait decline in aging, the present study used our previous protocol in two different conditions (walking and nonwalking chaotic plantar stimulations) in younger and older adults, to look for differential brain activation correlated with age during gait simulation. The aim was to determine whether the activation of specific brain areas involved in the integration of somatosensory information during walking changes with aging.

## 2 METHODS

### 2.1 Participants

#### 2.1.1 Recruitment

Healthy volunteers with no neurological or orthopedic disorders were recruited from the local clinical research center of the University Hospital of Angers (France) and included in the study from February 2015 to May 2017. All participants were right‐handed as confirmed by the Edinburgh Handedness Inventory for determining the dominant hand (Oldfield, 1971). The MMSE was carried out in all participants to confirm cognitive integrity, and minimal score for inclusion in the study was 27. It was also verified that patients did not have proprioceptive disorders by diapason testing. Inclusion criteria also included the absence of a history of stroke or other cerebral‐medullary pathology with sensory‐motor sequelae, the absence of depressive symptomatology, the absence of symptomatic peripheral neuropathy, or proprioceptive disorders. As the MMSE may underestimate patients with cognitive disorders in the early stages, the presence of a subjective cognitive complaint was an exclusion criteria. A part of our sample (18 healthy volunteers [7 women], aged from 20 to 40 years [mean 27 ± 4.7 years]) belonged to the previously published work already assessing brain areas associated with gait control (Labriffe et al., 2017). All volunteers were recruited during the same period and assessed with the same MRI unit for data acquisition.

#### 2.1.2 Ethics statement

The study was conducted in accordance with the ethical standards of the Helsinki Declaration (1983). Written informed consent was obtained at enrolment and the entire study protocol was approved by the University of Angers Ethical Review Committee (Comité de protection des personnes (CPP) ouest II, Angers, France, n° A.C. = 2014‐A01593‐44, n° CPP: 2014/32).

### 2.2 Korvit plantar pressure simulator

The MRI‐compatible Korvit simulator was used to mechanically stimulate the plantar support zones of the feet. As described in a previous publication (Labriffe et al., 2017), the Korvit system consists of a compressor connected by air cables to a pair of plastic boots (three sizes available) containing inflatable rubber chambers placed on the sole producing a pressure of 40 kPa on heel and toes load zones allowing dynamic foot stimulation simulating gait. The Korvit simulator was first developed by IBMP (Moscow, Russia) for cosmonauts to simulate walking in space and to reduce neuromuscular impairment following prolonged weightlessness (Layne & Forth, 2008). The device is manufactured by the companies “VIT” (Saint‐Petersburg, Russia) and “Center of Aviaspace medicine” (Moscow, Russia).

Two separate modes of stimulation were used in the present study: organized and chaotic. The organized mode produced gait‐like stimulation of the plantar surfaces of the feet, mimicking a cadence of 120 steps per minute. The cycle was as follows: right heel, right toes, left heel, left toes, and so on. The chaotic mode consisted of an apparently illogical (nongait‐like) pattern of stimulation: right heels, left toes, right toes, left heels, and so on. The cadence was similar to the organized mode, that is, 120 “pseudo”‐steps per minute.

The stimulation force was always the same and not adapted to the patient's weight. Before starting the acquisition, it was necessary to check that the patient perceived the pressures well.

### 2.3 Magnetic resonance imaging

#### 2.3.1 Preparation and data acquisition

Functional magnetic resonance imaging was performed for each volunteer on the same clinical 3T MRI unit (Magnetom Skyra, Siemens, Erlangen, Germany), using a standard transmitter‐receiver head coil. Participants laid comfortably in the machine, with headphones on to hear the instructions, foam blocks to keep the head still, and the Korvit boots on their feet. They looked through a prism at a screen positioned at their feet. Lights were turned off during image acquisition. Participants were strictly instructed not to move during the entire protocol. Surface EMG (3 MRI‐compatible EMG electrodes placed on the tibialis anterior and soleus muscles) was used to monitor activity, in live, through the Biopack device to check that participants did not perform any voluntary muscle contractions during the protocol. It was recorded for each subject. The motor activity was checked before each recording by performing voluntary movements.

A three‐dimensional high‐resolution T1‐weighted volume covering the whole brain was acquired (192 contiguous axial slices, 256 × 256 in‐plane matrix, yielding a voxel size of 1 mm × 1 mm × 1 mm), thereby providing an anatomical image for further co‐registration and normalization. An echo‐planar imaging sequence was used to acquire functional sessions for each participant (repetition time 2,280 ms, echo time 30 ms, flip angle 90°, 40 axial slices interleaved, 4.0 mm thick, 0 mm gap, in a 64 × 64 plane matrix, yielding a voxel size of 3.75 mm × 3.75 mm × 4 mm, field of view 240 mm), covering the whole brain, including the cerebellum. Three separate fMRI sessions, including 150 functional volumes per session, were performed for each participant during the same MRI procedure.

#### 2.3.2 Experimental design

The fMRI study was organized as a block‐design experiment. Each session involved two consecutive conditions. Each condition was performed for 19 s and repeated nine times, for a total session duration of 5 min 42 s. Two fMRI sessions were carried out for the present analysis.

Session #1 consisted of alternating an ORGANIZED condition and a REST_Organized_ condition. In the ORGANIZED condition, the Korvit boots were activated and produced a structured pattern of pressures, similar to the pattern of foot pressures during gait. The participant was instructed to look at a white cross in the middle of a black screen, and to remain perfectly still. During the REST_Organized_ condition, the participant continued to look at the cross, but the boots were disabled and no stimulation was applied.

Session #2, which was composed of a CHAOTIC condition and a REST_Chaotic_ condition, was organized just as session #1, except that Korvit boots were activated with a chaotic pattern which did not mimic foot pressures during gait.

### 2.4 Data treatment

#### 2.4.1 Image preprocessing

Functional Magnetic Resonance Imaging data were analyzed using SPM12 (Wellcome Department of Imaging Neuroscience, University College, London, UK) implemented on Matlab (The MathWorks, Natick, Massachusetts). First, native space images were corrected for the time delay between different slices (slice timing step). Then, they were realigned to the first volume and unwrapped to correct for head movements and susceptibility distortions. Participants were excluded from analysis if head motion was greater than 3 mm or greater than 3° during the whole fMRI session. Coregistration of images from different sessions was achieved using mean echo‐planar of slice‐timed and motion‐corrected unwrapped images as reference image, and 3D T1‐weighted anatomical image as source image. The 3D T1 volume was segmented in native‐space, using a unified segmentation approach (Ashburner & Friston, 2005). Echo‐planar images were rewritten to a final resolution of 3 mm × 3 mm × 3 mm and normalized to the Montreal Neurological Institute (Collins, Neelin, Peters, & Evans, 1994) template (MNI template) using the forward deformation field generated during segmentation. Finally, functional images were smoothed by an isotropic Gaussian kernel of 8 mm full‐width at half‐maximum.

#### 2.4.2 fMRI statistical analysis

First level statistical analysis was carried out for each participant by modeling the different conditions as separate regressors in the same general linear model (GLM) (Friston et al., 1995). A highpass filter with a cut‐off of 128 s was used to remove low frequency noise.

Each individual specific design matrix was filled with the following condition order: CHAOTIC, REST_Chaotic_, ORGANIZED, REST_Organized_. To reduce artifacts from participant movements, the alignment rigid transformation parameters were also introduced as regressors.

Thus, three contrast images were computed with the following vectors: (a) ORGANIZED > REST_Organized_; (b) CHAOTIC > REST_Chaotic_; (c) (ORGANIZED > REST_Organized_) > (CHAOTIC > REST_Chaotic_).

In order to make broader inferences about the general population from which the participants were drawn, each participant's contrast images from the first level analysis were entered into a random effects second‐level analysis using multiple regressions with age as covariate and gender as confounding factor.

Hence, the resulting statistical parametric maps were analyzed by multiple‐factor regression conducted under standard cluster‐extent based thresholding methods. The regression factors were defined as age and gender. Voxels with a *p* <.001 as proposed in Woo, Krishnan, and Wager (2014) and cluster size (*k*) > 38 voxels were considered as showing a significant correlation with age. That threshold was determined by the Monte‐Carlo simulation *(*Woo et al., *2014* *)* after correction for multiple comparisons (Ledberg, Akerman, & Roland, 1998). This was performed by the program REST AlphaSim (Song et al., 2011) which is based on AlphaSim in AFNI (Ward, 2000). Of note, input parameters to AlphaSim as implemented in the SPM REST toolbox included an individual voxel threshold probability of *p* < .001, cluster connection radius of 5 mm, and 8 mm full width at half maximum smoothness. Thus, the estimated minimum cluster size extent was defined as 38 voxels for the SPM map in order to satisfy a family‐wise error rate correction of *p* < .05.

Anatomical correlates of clusters of activation were determined visually and with the help of probabilistic cytoarchitectonic maps implemented in the Anatomy toolbox (Eickhoff et al., 2005).

## 3 RESULTS

### 3.1 Participants

Sixty‐seven healthy volunteers (35 women), aged between 20 and 77 years (mean ± standard deviation: 49.2 ± 18.0 years), were included in the study after verification of the compliance with the inclusion or exclusion criteria. A subset of our sample (18 healthy volunteers [7 women] aged from 20 to 40 years [27 ± 4.7 years]) took part in a previous study assessing brain areas associated with gait control (Labriffe et al., 2017). Due to excessive head movements, two subjects were excluded from the analyses (1 subject <40 years, 0 subjects 40–60 years, 1 subject >60 years).

Participant cognitive integrity was confirmed by a Mini Mental State Examination (score > 27). The EMG recording did not show any calf contractions.

The age of participants is described in Table 1.

**TABLE 1:** Age distribution of included volunteers

Age (years)	18–28	28–38	38–48	48–58	58–68	68–78
*n*	14	6	12	11	14	10

### 3.2 Age‐related study analysis

In order to investigate the potential correlation between age and/or gender and activation zones, a two‐step analysis was necessary: (a) analysis of the individual data and (b) multiple regressions. Results of the multiple regression analysis were the main focus of this study and allowed to identify brain areas showing a correlation between the activation/deactivation intensity and the age of the subjects.

Concerning the individual data, we performed a statistical analysis of the ORGANIZED > REST_Oraganized_ and CHAOTIC > REST_Chaotic_ contrasts over the entire population (67 subjects) and among three subgroups (18–38 years, 20 subjects; 38–58 years, 23 subjects; 58–78 years, 24 subjects). For clarity, these primary activation data are not detailed in this manuscript but are available as Supporting Information (Appendix S1). These results essentially match with the activations found in our previous study (Labriffe et al., 2017).

**FIGURE 1:**
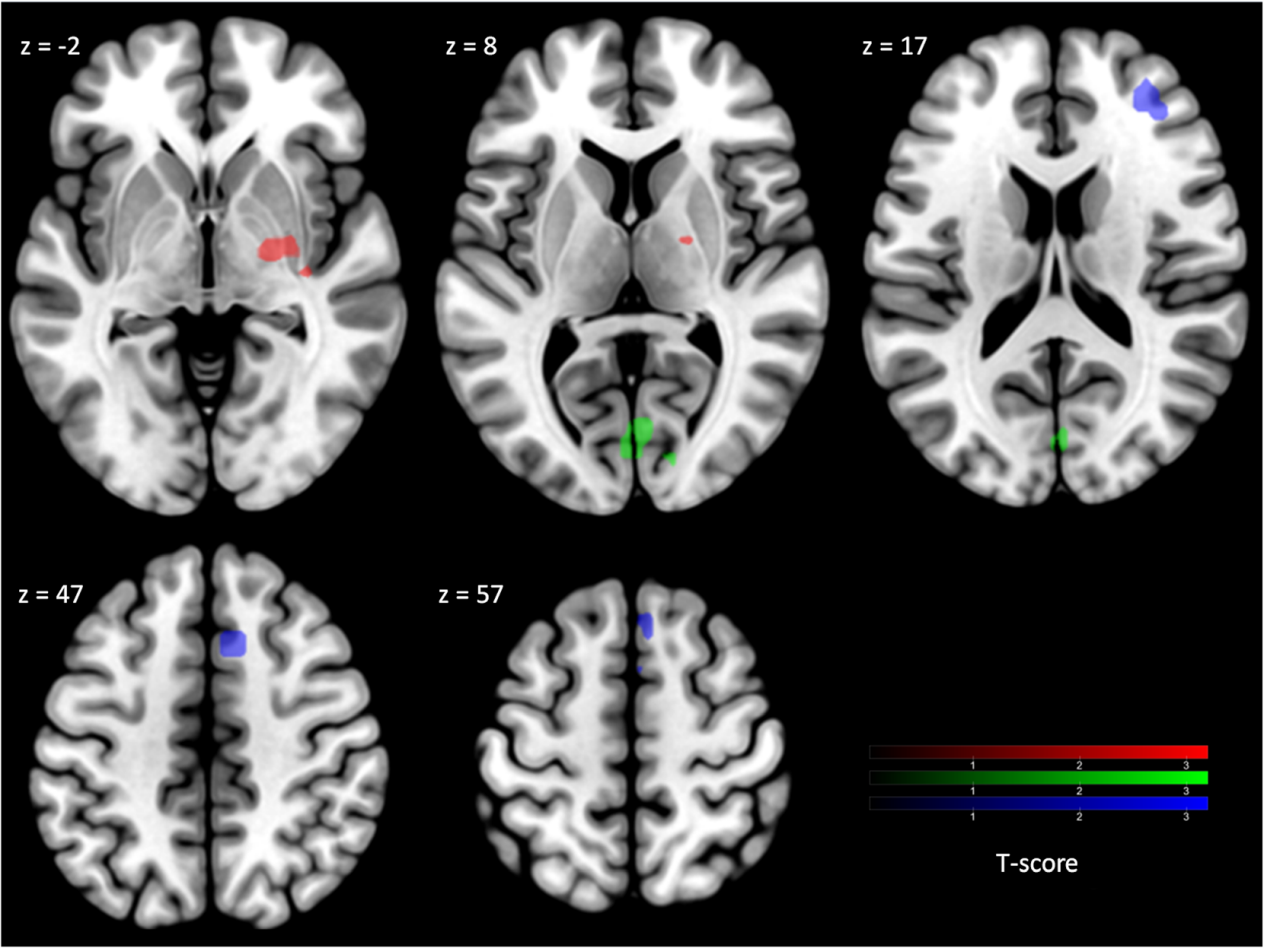
Statistical parametric map for the multiple regressions of age‐related contrasts with voxel level uncorrected *p* < .001, cluster‐size threshold 38 voxels, satisfying a family‐wise error rate correction of *p* < .05 as assessed by Monte‐Carlo method. Red areas correspond to positive correlation on the ORGANIZED > REST_Organized_ contrast; green areas to negative correlation on the CHAOTIC > REST_Chaotic_; blue areas to positive correlation on the (ORGANIZED > REST_Organized_) > (CHAOTIC > REST_Chaotic_) contrast. Images are presented according to the neurological convention (right brain on the right)

Concerning multiple regression, an overview of our main results, is shown in Figure 1. All statistical maps were generated with a threshold providing a family‐wise error rate correction of *p* < .05. Of note, there was no specific influence of gender (as confounding factor) on activation/deactivation zones.

**TABLE 2:** Significant clusters and their corresponding maxima for the correlation between the ORGANIZED > REST_Organized_, CHAOTIC > REST_Chaotic_, and (ORGANIZED > REST_Organized_) > (CHAOTIC > REST_Chaotic_) contrasts and age

Cluster #	Voxels	Anatomical region		Side	MNI coordinates (mm) of the peak			*T* score
					*x*	*y*	*z*	
(a) ORGANIZED > REST_Organized_: *Positive* correlation with age								
1	39	Pallidum		R	24	−10	−1	4.38
		Pre‐motor thalamus		R	21	−7	8	3.77
		Putamen		R	33	−19	−4	3.31
(b) CHAOTIC > REST_Chaotic_: *Negative* correlation with age								
1	68	V1	BA17	R	3	−76	8	3.91
		V1	BA17	L	0	−82	11	3.78
(c) (ORGANIZED > REST_Organized_) > (CHAOTIC > REST_Chaotic_): *Positive* correlation with age								
1	60	Pre‐SMA	BA6	R	6	26	62	4.94
		F1M—DLPFC	BA8	R	12	17	47	4.85
2	50	F2—Mid‐DLPFC	BA9	R	39	38	20	4.13
		F2—Mid‐DLPFC	BA10	R	33	44	17	3.90

*Note*: Voxel level uncorrected *p* < .001, cluster‐size threshold 38 voxels, satisfying a family‐wise error rate correction of *p* < .05 as assessed by Monte‐Carlo method; *x*,*y*,*z*: original SPM coordinates of the MNI space; in case of multiple peaks in the same anatomic area of a cluster, only the maximal peak is reported.

Abbreviations: BA, Brodmann area; DLPFC, dorsolateral prefrontal cortex; F1M, medial part of the superior frontal gyrus; F2, middle frontal gyrus; SMA, supplementary motor area; V1, primary visual cortex.

#### 3.2.1 Brain age‐correlated activation during gait‐like plantar stimulation

The multiple regression applied on the ORGANIZED > REST_Oraganized_ contrast shows:

1. A *positive* correlation between age and brain activation in one cluster extended over right pallidum, thalamus (more precisely its premotor and prefrontal connected areas) and putamen, and covering white matter in the posterior limb of the right internal capsule, which means these regions were significantly (and proportionally) more activated with increasing age in healthy subjects. Details on locations are given in Table 2a.
2. No *negative* correlation between age and brain activations.

#### 3.2.2 Brain age‐correlated activation during chaotic plantar stimulation

The multiple regression applied on the CHAOTIC > REST_Chaotic_ contrast shows:

1. No *positive* correlation between age and brain activations;
2. A *negative* correlation between age and brain activation in one cluster centered on the bilateral occipital cortex. The cluster expands over the upper and medium side of both right and left calcarine areas (primary visual area V1, Brodmann area [BA] 17), with a small extension downward and forward on the secondary visual areas (V2, BA18), which means these regions were significantly (and proportionally) less activated with increasing age in healthy subjects. Details on locations are shown in Table 2b

#### 3.2.3 Brain age‐correlated differential activation between gait‐like versus chaotic plantar stimulation

**FIGURE 2:**
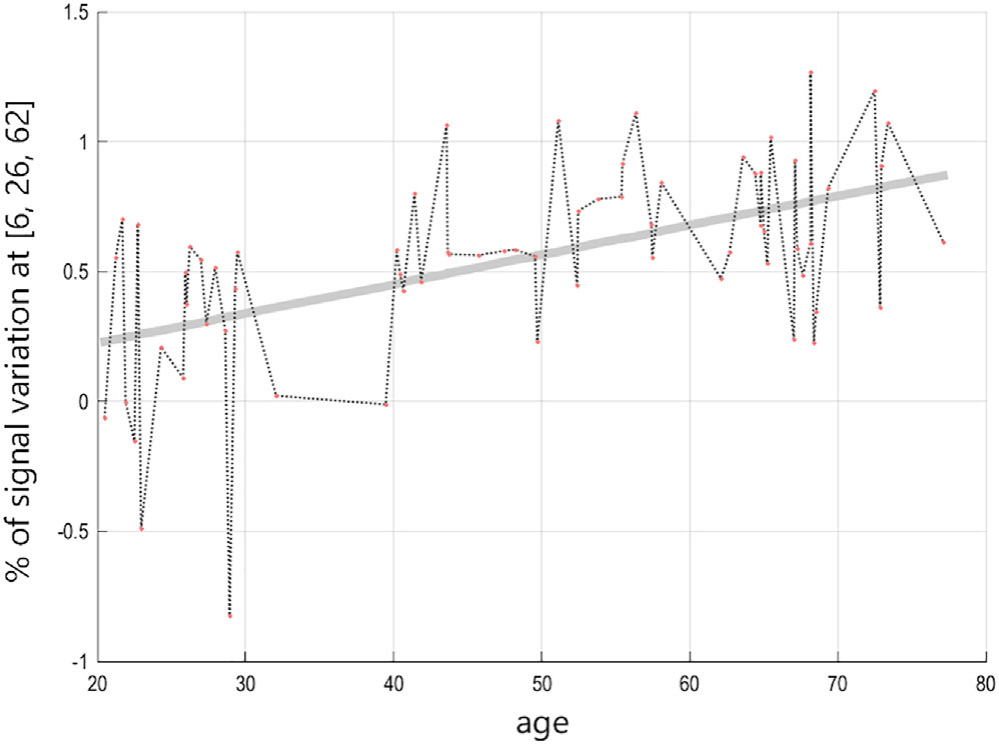
Representative regression curve on statistical activation parameters centered on the main peak (MNI location 6, 26, 62) of age‐correlated activation on the (ORGANIZED > REST_Organized_) > (CHAOTIC > REST_Chaotic_) contrast. Response represents the relative activation on the considered MNI location (*x*,*y*,*z*) for each participant depending on its age. The curve in gray shows the predictive response build from the correlation parameters

The multiple regression applied on the (ORGANIZED > REST_Organized_) > (CHAOTIC > REST_Chaotic_) contrast shows:

1. A *positive* correlation between age and brain activation in two clusters, which means these regions were significantly (and proportionally) more activated with increasing age in healthy subjects. Cluster #1 is centered on the most anterior part of right supplementary motor area (SMA, BA6), known as pre‐SMA, with a forward extension to the medial part of the superior frontal gyrus (F1M, BA8) and a downward extension in the vicinity of the anterior rostral cingulate area. Cluster #2 is centered on anterior part of right middle frontal gyrus (F2) and spans on both BA9 et BA10, in a region known as mid‐dorsolateral prefrontal precortex (mid‐DLFPC) (Badre & Nee, 2018). Details on locations are given in Table 2c. A representative correlation curve for the main age‐correlated activation peaks is shown in Figure 2.
2. No *negative* correlation between age and brain activations.

## 4 DISCUSSION

This fMRI study was designed to determine whether the level of brain activation of specific areas involved in the integration of somatosensory information in the walking sequence is correlated with age. Gait was simulated in the fMRI setting by using a purely mechanical plantar stimulation. Two conditions were studied separately: an organized plantar stimulation simulating real gait and a chaotic plantar stimulation that generated a mechanical stimulus that could not be integrated by the brain as gait‐related. Then, the comparison between these two conditions was used to isolate the gait‐specific plantar stimulation pattern (signal) from the confounding mechanical somatosensory stimulus (noise).

The analysis of the first condition tested (contrast ORGANIZED > REST_Organized_) showed age‐correlated activation of right pallidum, thalamus (pre‐motor and prefrontal areas), and putamen. The analysis of the second condition (contrast CHAOTIC > REST_Chaotic_) showed age inversely correlated activation of bilateral primary visual cortex (V1). The analysis of the difference between the first and the second condition (contrast (ORGANIZED > REST_Organized_) > (CHAOTIC > REST_Chaotic_)) showed age correlated activation of right pre‐SMA (BA6 with a small extension to the border of BA8) and mid‐dorsolateral prefrontal cortex (mid‐DLPFC, BA9‐10). Of note, no other area of age‐correlated over‐ or deactivation was found in this study, which shows the specificity of aforementioned areas. These results are consistent with previous neuroimaging studies on the impact of aging on gait performance (Allali et al., 2014; Holtzer et al., 2014).

This analysis was based on multiple regressions of individual data in relation to age (with gender as potential confounding factor). This approach results in a biological gradient (fifth criteria of Hill, see Hill [1965]) which is very conservative, as compared to an analysis based on the comparison of groups of participants with different age ranges. This is probably the reason why among the numerous activated areas previously shown in Labriffe et al. (2017), only few and limited areas were positive for age correlation in this study, even with the relatively high power due to the inclusion of a high number of participants as compared to other fMRI studies (Holtzer et al., 2014). To the best of our knowledge, the present study is the first one to investigate the integration of somatosensory information in gait evolution during advance in age using multiple regression regardless of the neuroimaging technique used (Holtzer et al., 2014; Wilson et al., 2019). Consequently, our study makes it possible to: (a) confirm, through a robust study (both in terms of size and methodology), the brain areas involved in age‐related changes in somatosensory processing of gait; and (b) support the use, in older adults, of plantar simulation in a simulated gait model for the identification of neural correlates of gait in a fMRI setting.

The three areas involved in the gait‐related stimulation are key regions of the thalamocortical‐basal ganglia loop that controls gait at the subcortical level (Jahn et al., 2008). These activations are found in the majority of neuroimaging studies on gait control in aging. To date, most of these studies were conducted with small samples, and their results were presented as hypothetical. The robustness of the methodology used in the present study, and the consistency of the results with previous studies now makes it possible to confirm these hypotheses.

The activation of primary visual cortex in younger subjects in the second contrast can be more surprising, as far as no visual stimulation was applied during acquisition (participants were instructed to remain still while looking at a white cross in the center of a black screen). An unusual plantar stimulation could thus lead younger subjects to activate and focus on another sensory input (sight), whereas older subjects were less distracted by such a stimulus (Zwergal et al., 2012). As far as visual perception and visual imagination both involve primary visual cortex (Ganis, Thompson, & Kosslyn, 2004), we could also understand V1 activation as an intent to visualize the situation responsible for the uncommon plantar sensation, which could be more pronounced in younger subjects.

Visual information is a crucial sensory input for safe walking. Diminished or abnormal visual input has been associated with a decreased performance in tasks testing the control of steady state walking (Helbostad, Vereijken, Hesseberg, & Sletvold, 2009; Swenor, Muñoz, & West, 2014) and more complex tasks like obstacle crossing and curb negotiation (Alexander et al., 2014a, 2014b; Novak & Deshpande, 2014). Lack of visual information has been proposed as an important factor leading to compromised automaticity of walking (Clark, 2015), and this phenomenon may be more pronounced in older adults with less activation of primary visual cortex than younger subjects.

The SMA is well known for its involvement in motor control. This region occupies the posterior third of the superior frontal gyrus (corresponding to BA6) and is a functionally distinct premotor area thought to operate at a hierarchically superior level to that of the precentral motor cortex. For example, SMA has been involved in imagined movements and its activation is increased in more complex tasks (Roland, Larsen, Lassen, & Skinhøj, 1980). A landmark review by Picard and Strick (1996) introduced the concept of SMA being divided in two distinct motor areas: pre‐SMA (rostral to a coronal plane located on the anterior commissure) and SMA proper (caudal to this plane). The authors reviewed almost 30 PET studies addressing its functional activation and showed a caudo‐rostral gradient of SMA activation with increasing task complexity. In these studies, pre‐SMA activation was linked to complex, unfamiliar tasks, learning, and decision, whereas SMA proper activation was related to usual, automated tasks, practice and skill. Hence, by exhibiting an age‐correlated activation of the most anterior part of pre‐SMA, our results show that gait tends to be treated as a more complex task in older adults.

Even more interesting is the involvement of right mid‐DLPFC. Our comprehension of the prefrontal region remains elusive. Executive functions have generally included abilities related to goal formation, planning, carrying out goal‐directed plans and effective performance, and these wide tasks include many subtasks that have been listed in Jurado and Rosselli (2007). It is still unclear whether there is one single underlying ability that can explain all the components of executive functions (also known as the theory of unity) (Koechlin et al., 2003) or whether these components constitute related but distinct task processes (nonunity), because there seems to be evidence for both unitary and nonunitary nature of executive function (Jurado & Rosselli, 2007). The hypothesis of a rostro‐caudal organization of DLPFC's function according to a unidimensional abstraction gradient has been proposed (Badre, 2008). Alternatively, separate frontal networks could interact via local and global hierarchical structure to support diverse task demands, with mid‐DLPFC as the top of the frontal hierarchy (Badre & Nee, 2018). Of note, the involvement of DLPFC in the present study appeared right‐sided, which is consistent with the notion that right DLPFC is more involved in monitoring behavior while the left DLPFC is more involved in verbal processing (Kandel, Schwartz, Jessell, Siegelbaum, & Hudspeth, 2012).

All these theories share the idea that mid‐DLPFC, and the prefrontal cortex in general, operate at the highest levels of the control hierarchy, as they contribute to the cascade of processes that mediate task planning and execution of cognitive and motor functions, and play an essential role as an interface between cognition, action and physical world (Clark, 2015). For example, and not exhaustively, prefrontal cortical activity is heightened during the performance of cognitive tasks (Herrmann, Walter, Ehlis, & Fallgatter, 2006), fine motor tasks (Okamoto et al., 2004), decision‐making task (Tannou, Magnin, Comte, Aubry, & Joubert, 2021), and dual‐tasks (Roee Holtzer et al., 2011). Nevertheless, it can also be considered that a higher activation of BA9‐10 corresponds to response inhibition (e.g., to prevent an actual muscle movement), especially given that cortical compensation is increased during aging. Prefrontal cortical activation has been correlated with complexity of both cognitive (Shibuya‐Tayoshi et al., 2007) and walking (Clark, Rose, Ring, & Porges, 2014) tasks, or with gait initiation (Suzuki et al., 2004), whereas steady state walking is undemanding (Suzuki et al., 2004).

Our results can be interpreted as a clue for a progressive age‐related increase in the involvement of executive functions during walking, and especially the more hierarchical ones, supporting a continuous process of compromised automaticity as proposed in Clark (2015). In the present study, we identified specific locations related to this process whose activation could be evaluated in real gait, to assess the risk of falling (Fernandez, Hars, Trombetti, & Vuilleumier, 2019) or to evaluate cognitive decline, since gait assessment could be crucial in the early diagnosis of dementia or predementia (Verghese et al., 2002, 2008; Verghese, Wang, Lipton, Holtzer, & Xue, 2007).

This passive peripheral stimulation tool to study gait control in older people is an innovative approach based on preliminary studies carried out by our team. This research has resulted in a proof of concept with younger adults (Labriffe et al., 2017). As previously described, the study of gait control with neuroimaging techniques involves a wide range of tools that are used to compensate for the impossibility of real gait, particularly in fMRI. Among these tools, the most commonly used are imagined walking or walking simulated by a video during imaging. Nevertheless, although they provide good results, these processes remain limited. In particular, since prefrontal cognitive activation suggests an impairment of automatic gait control in aging, the demonstration of increased activation in double‐task tests is relevant as a predictive tool for gait disorders. It could also be used as a predictive marker of the risk of frailty. To confirm this link between increased activations, gait disorders and risk of frailty, it would be necessary to validate a method combining peripheral simulation, gait in ecological settings (as much as possible), and complex analysis of dual task paradigms. Since our study confirmed the known patterns of gait activation in aging, as previously identified by reference techniques, it supports the validity of our hypothesis, and thus the use of peripheral stimulation as a tool to assess gait‐related activity. Ultimately, the development of this tool could be very useful for the analysis of gait associated with neurocognitive disorders. Gait alterations could thus be studied through a passive approach, which distinguishes this strategy from other tools developed in peripheral simulation (Bürki et al., 2017).

Nevertheless, our model could probably be optimized by adjusting plantar stimulation pressure or step rate (Gonzales, Al‐Khalil, & O'Boyle, 2019), or the boots could be improved to increase the spatial resolution of the pressure being applied. Our model was basic: there were no start or end points, direction, speed, or any visual information that may have increased the realism of gait sensations, and this may have complicated the task, adding possible confounding factors, or unraveling activation areas linked to these special conditions rather than gait.

## 5 CONCLUSIONS

To the best of our knowledge, this study addresses for the first time the intriguing question of the evolution of gait‐related brain activation with aging by using multiple regressions in a broad population of cognitively healthy younger and older adults. Our study also presents the particularity to validate the use of a plantar simulation system to analyze the gait control using fMRI. Our results showed specific age‐correlated activity in various areas that are known to be involved in gait control, strengthening the relevance of our simulation model as a useful and reproducible tool for functional gait study. We also highlighted a gradual and specific activation of top hierarchical cortical motor control areas in simulated gait that are associated with aging, supporting a progressive loss of automation in older adults.

## CONFLICT OF INTEREST

The authors declare that there is no potential conflict of interest.

## AUTHORS' CONTRIBUTIONS

The “IRMarche” study was conceived by Mickaël Dinomais. The analytical framework was designed by Matthieu Labriffe, under the supervision of Mickaël Dinomais, and performed by Henry Jeanvoine and Matthieu Labriffe under the supervision of Mickaël Dinomais. The article was written by Henry Jeanvoine under the supervision of Matthieu Labriffe and Mickaël Dinomais. All authors contributed to data collection and interpretation. All authors revised and critically appraised the intellectual content of the manuscript, and approved the final version.

## Supporting information


**Appendix** **S1**: Supporting information

## Data Availability

The data that support the findings of this study are available on request from the corresponding author, ML. The data are not publicly available since they contain information that could compromise the privacy of research participants.

## References

[hbm25691-bib-0001] Åkerborg, Ö. , Lang, A. , Wimo, A. , Sköldunger, A. , Fratiglioni, L. , Gaudig, M. , & Rosenlund, M. (2016). Cost of dementia and its correlation with dependence. Journal of Aging and Health, 28(8), 1448–1464. 10.1177/0898264315624899 26818587

[hbm25691-bib-0002] Alexander, M. S. , Lajoie, K. , Neima, D. R. , Strath, R. A. , Robinovitch, S. N. , & Marigold, D. S. (2014a). Effect of ambient light and age‐related macular degeneration on precision walking. Optometry and Vision Science: Official Publication of the American Academy of Optometry, 91(8), 990–999. 10.1097/OPX.0000000000000316 24987813

[hbm25691-bib-0003] Alexander, M. S. , Lajoie, K. , Neima, D. R. , Strath, R. A. , Robinovitch, S. N. , & Marigold, D. S. (2014b). Effects of age‐related macular degeneration and ambient light on curb negotiation. Optometry and Vision Science: Official Publication of the American Academy of Optometry, 91(8), 975–989. 10.1097/OPX.0000000000000286 24879086

[hbm25691-bib-0004] Allali, G. , van der Meulen, M. , Beauchet, O. , Rieger, S. W. , Vuilleumier, P. , & Assal, F. (2014). The neural basis of age‐related changes in motor imagery of gait: An fMRI study. The Journals of Gerontology: Series A, 69(11), 1389–1398. 10.1093/gerona/glt207 24368777

[hbm25691-bib-0005] Ashburner, J. , & Friston, K. J. (2005). Unified segmentation. NeuroImage, 26(3), 839–851. 10.1016/j.neuroimage.2005.02.018 15955494

[hbm25691-bib-0006] Axer, H. , Axer, M. , Sauer, H. , Witte, O. W. , & Hagemann, G. (2010). Falls and gait disorders in geriatric neurology. Clinical Neurology and Neurosurgery, 112(4), 265–274. 10.1016/j.clineuro.2009.12.015 20089351

[hbm25691-bib-0007] Badre, D. (2008). Cognitive control, hierarchy, and the rostro‐caudal organization of the frontal lobes. Trends in Cognitive Sciences, 12(5), 193–200. 10.1016/j.tics.2008.02.004 18403252

[hbm25691-bib-0008] Badre, D. , & Nee, D. E. (2018). Frontal cortex and the hierarchical control of behavior. Trends in Cognitive Sciences, 22(2), 170–188. 10.1016/j.tics.2017.11.005 29229206 PMC5841250

[hbm25691-bib-0009] Blumen, H. M. , Holtzer, R. , Brown, L. L. , Gazes, Y. , & Verghese, J. (2014). Behavioral and neural correlates of imagined walking and walking‐while‐talking in the elderly. Human Brain Mapping, 35(8), 4090–4104. 10.1002/hbm.22461 24522972 PMC4106989

[hbm25691-bib-0010] Bürki, C. N. , Bridenbaugh, S. A. , Reinhardt, J. , Stippich, C. , Kressig, R. W. , & Blatow, M. (2017). Imaging gait analysis: An fMRI dual task study. Brain and Behavior, 7(8), e00724. 10.1002/brb3.724 28828204 PMC5561304

[hbm25691-bib-0011] Clark, D. J. (2015). Automaticity of walking: Functional significance, mechanisms, measurement and rehabilitation strategies. Frontiers in Human Neuroscience, 9, 246. 10.3389/fnhum.2015.00246 25999838 PMC4419715

[hbm25691-bib-0012] Clark, D. J. , Rose, D. K. , Ring, S. A. , & Porges, E. C. (2014). Utilization of central nervous system resources for preparation and performance of complex walking tasks in older adults. Frontiers in Aging Neuroscience, 6, 217. 10.3389/fnagi.2014.00217 25202270 PMC4142860

[hbm25691-bib-0013] McCrimmon, C. M. , Wang, P. T. , Heydari, P. , Nguyen, A. , Shaw, S. J. , Gong, H. , … Do, A. H. (2018). Electrocorticographic encoding of human gait in the leg primary motor cortex. Cerebral Cortex, 28(8), 2752–2762. 28981644 10.1093/cercor/bhx155 PMC6248549

[hbm25691-bib-0014] Collins, D. L. , Neelin, P. , Peters, T. M. , & Evans, A. C. (1994). Automatic 3D intersubject registration of MR volumetric data in standardized Talairach space. Journal of Computer Assisted Tomography, 18(2), 192–205. 8126267

[hbm25691-bib-0015] Eickhoff, S. B. , Stephan, K. E. , Mohlberg, H. , Grefkes, C. , Fink, G. R. , Amunts, K. , & Zilles, K. (2005). A new SPM toolbox for combining probabilistic cytoarchitectonic maps and functional imaging data. NeuroImage, 25(4), 1325–1335. 10.1016/j.neuroimage.2004.12.034 15850749

[hbm25691-bib-0016] Fernandez, N. B. , Hars, M. , Trombetti, A. , & Vuilleumier, P. (2019). Age‐related changes in attention control and their relationship with gait performance in older adults with high risk of falls. NeuroImage, 189, 551–559. 10.1016/j.neuroimage.2019.01.030 30660655

[hbm25691-bib-0017] Friston, K. J. , Holmes, A. P. , Worsley, K. J. , Poline, J. P. , Frith, C. D. , & Frackowiak, R. S. J. (1995). Statistical parametric maps in functional imaging: A general linear approach. Human Brain Mapping, 2, 189–210. 10.1002/hbm.460020402

[hbm25691-bib-0018] Ganis, G. , Thompson, W. L. , & Kosslyn, S. M. (2004). Brain areas underlying visual mental imagery and visual perception: An fMRI study. Cognitive Brain Research, 20(2), 226–241. 10.1016/j.cogbrainres.2004.02.012 15183394

[hbm25691-bib-0019] Godde, B. , & Voelcker‐Rehage, C. (2010). More automation and less cognitive control of imagined walking movements in high‐ versus low‐fit older adults. Frontiers in Aging Neuroscience, 2, 139. 10.3389/fnagi.2010.00139 PMC2944669 20877433

[hbm25691-bib-0020] Gonzales, J. U. , Al‐Khalil, K. , & O'Boyle, M. (2019). Spatial task‐related brain activity and its association with preferred and fast pace gait speed in older adults. Neuroscience Letters, 713, 134526. 10.1016/j.neulet.2019.134526 31585208 PMC6858846

[hbm25691-bib-0021] Grande, G. , Triolo, F. , Nuara, A. , Welmer, A.‐K. , Fratiglioni, L. , & Vetrano, D. L. (2019). Measuring gait speed to better identify prodromal dementia. Experimental Gerontology, 124, 110625. 10.1016/j.exger.2019.05.014 31173841

[hbm25691-bib-0022] Grillner, S. (2006). Biological pattern generation: The cellular and computational logic of networks in motion. Neuron, 52(5), 751–766. 10.1016/j.neuron.2006.11.008 17145498

[hbm25691-bib-0023] Harada, C. N. , Natelson Love, M. C. , & Triebel, K. L. (2013). Normal cognitive aging. Clinics in Geriatric Medicine, 29(4), 737–752. 10.1016/j.cger.2013.07.002 24094294 PMC4015335

[hbm25691-bib-0024] Heinrich, S. , Rapp, K. , Rissmann, U. , Becker, C. , & König, H.‐H. (2010). Cost of falls in old age: A systematic review. Osteoporosis International: A Journal Established as Result of Cooperation between the European Foundation for Osteoporosis and the National Osteoporosis Foundation of the USA, 21(6), 891–902. 10.1007/s00198-009-1100-1 19924496

[hbm25691-bib-0025] Helbostad, J. L. , Vereijken, B. , Hesseberg, K. , & Sletvold, O. (2009). Altered vision destabilizes gait in older persons. Gait & Posture, 30(2), 233–238. 10.1016/j.gaitpost.2009.05.004 19487126

[hbm25691-bib-0026] Herrmann, M. J. , Walter, A. , Ehlis, A.‐C. , & Fallgatter, A. J. (2006). Cerebral oxygenation changes in the prefrontal cortex: Effects of age and gender. Neurobiology of Aging, 27(6), 888–894. 10.1016/j.neurobiolaging.2005.04.013 16023767

[hbm25691-bib-0027] Hill, A. B. (1965). The environment and disease: Association or causation. Proceedings of the Royal Society of Medicine, 58, 295–300. 14283879 10.1177/003591576505800503 PMC1898525

[hbm25691-bib-0028] Holtzer, R. , Epstein, N. , Mahoney, J. R. , Izzetoglu, M. , & Blumen, H. M. (2014). Neuroimaging of mobility in aging: A targeted review. The Journals of Gerontology Series A: Biological Sciences and Medical Sciences, 69(11), 1375–1388. 10.1093/gerona/glu052 PMC4204614 24739495

[hbm25691-bib-0029] Holtzer, R. , Mahoney, J. R. , Izzetoglu, M. , Izzetoglu, K. , Onaral, B. , & Verghese, J. (2011). fNIRS study of walking and walking while talking in young and old individuals. The Journals of Gerontology, Series A, Biological Sciences and Medical Sciences, 66(8), 879–887. 10.1093/gerona/glr068 PMC3148759 21593013

[hbm25691-bib-0030] Jahn, K. , Deutschländer, A. , Stephan, T. , Kalla, R. , Hüfner, K. , Wagner, J. , … Brandt, T. (2008). Supraspinal locomotor control in quadrupeds and humans. Progress in Brain Research, 171, 353–362. 10.1016/S0079-6123(08)00652-3 18718326

[hbm25691-bib-0031] Jeannerod, M. (1995). Mental imagery in the motor context. Neuropsychologia, 33(11), 1419–1432. 8584178 10.1016/0028-3932(95)00073-c

[hbm25691-bib-0032] Jurado, M. B. , & Rosselli, M. (2007). The elusive nature of executive functions: A review of our current understanding. Neuropsychology Review, 17(3), 213–233. 10.1007/s11065-007-9040-z 17786559

[hbm25691-bib-0033] Kandel, E. R. , Schwartz, J. H. , Jessell, T. M. , Siegelbaum, S. A. , & Hudspeth, A. J. (2012). Chapter 19: Integration of sensory and motor function: The association areas of the cerebral cortex and the cognitive capabilities of the brain. In Principles of neural science (5th ed., p. 364). New York, NY: McGraw‐Hill Education/Medical.

[hbm25691-bib-0034] Koechlin, E. , Ody, C. , & Kouneiher, F. (2003). The architecture of cognitive control in the human prefrontal cortex. Science (New York, N.Y.), 302(5648), 1181–1185. 10.1126/science.1088545, 302, 1181, 1185 14615530

[hbm25691-bib-0035] Kremneva, E. I. , Chernikova, L. A. , Konovalov, R. N. , Krotenkova, M. V. , Saenko, I. V. , & Kozlovskaia, I. B. (2012). Activation of the sensorimotor cortex with the use of a device for the mechanical stimulation of the plantar support zones. Fiziologiia Cheloveka, 38(1), 61–68. 22567837

[hbm25691-bib-0036] la Fougère, C. , Zwergal, A. , Rominger, A. , Förster, S. , Fesl, G. , Dieterich, M. , … Jahn, K. (2010). Real versus imagined locomotion: A [18F]‐FDG PET‐fMRI comparison. NeuroImage, 50(4), 1589–1598. 10.1016/j.neuroimage.2009.12.060 20034578

[hbm25691-bib-0037] Labriffe, M. , Annweiler, C. , Amirova, L. E. , Gauquelin‐Koch, G. , Ter Minassian, A. , Leiber, L.‐M. , … Dinomais, M. (2017). Brain activity during mental imagery of gait versus gait‐like plantar stimulation: A novel combined functional MRI paradigm to better understand cerebral gait control. Frontiers in Human Neuroscience, 11, 106. 10.3389/fnhum.2017.00106 28321186 PMC5337483

[hbm25691-bib-0038] Layne, C. S. , & Forth, K. E. (2008). Plantar stimulation as a possible countermeasure to microgravity‐induced neuromotor degradation. Aviation, Space, and Environmental Medicine, 79(8), 787–794. 10.3357/asem.2293.2008 18717120

[hbm25691-bib-0039] Le Ray, D. , Juvin, L. , Ryczko, D. , & Dubuc, R. (2011). Chapter 4—Supraspinal control of locomotion: The mesencephalic locomotor region. Progress in Brain Research, 188, 51–70. 10.1016/B978-0-444-53825-3.00009-7 21333802

[hbm25691-bib-0040] Ledberg, A. , Akerman, S. , & Roland, P. E. (1998). Estimation of the probabilities of 3D clusters in functional brain images. NeuroImage, 8(2), 113–128. 10.1006/nimg.1998.0336 9740755

[hbm25691-bib-0041] Newman, A. B. , Simonsick, E. M. , Naydeck, B. L. , Boudreau, R. M. , Kritchevsky, S. B. , Nevitt, M. C. , … Harris, T. B. (2006). Association of long‐distance corridor walk performance with mortality, cardiovascular disease, mobility limitation, and disability. JAMA, 295(17), 2018–2026. 10.1001/jama.295.17.2018 16670410

[hbm25691-bib-0042] Novak, A. C. , & Deshpande, N. (2014). Effects of aging on whole body and segmental control while obstacle crossing under impaired sensory conditions. Human Movement Science, 35, 121–130. 10.1016/j.humov.2014.03.009 24746603

[hbm25691-bib-0043] Okamoto, M. , Dan, H. , Shimizu, K. , Takeo, K. , Amita, T. , Oda, I. , … Dan, I. (2004). Multimodal assessment of cortical activation during apple peeling by NIRS and fMRI. NeuroImage, 21(4), 1275–1288. 10.1016/j.neuroimage.2003.12.003 15050555

[hbm25691-bib-0044] Oldfield, R. C. (1971). The assessment and analysis of handedness: The Edinburgh inventory. Neuropsychologia, 9(1), 97–113. 5146491 10.1016/0028-3932(71)90067-4

[hbm25691-bib-0045] Paraskevoudi, N. , Balcı, F. , & Vatakis, A. (2018). “Walking” through the sensory, cognitive, and temporal degradations of healthy aging. Annals of the New York Academy of Sciences., 1426, 72–92. 10.1111/nyas.13734 29741265

[hbm25691-bib-0046] Picard, N. , & Strick, P. L. (1996). Motor areas of the medial wall: A review of their location and functional activation. Cerebral Cortex (New York, NY: 1991), 6(3), 342–353. 10.1093/cercor/6.3.342 8670662

[hbm25691-bib-0047] Porro, C. A. , Francescato, M. P. , Cettolo, V. , Diamond, M. E. , Baraldi, P. , Zuiani, C. , … di Prampero, P. E. (1996). Primary motor and sensory cortex activation during motor performance and motor imagery: A functional magnetic resonance imaging study. The Journal of Neuroscience: The Official Journal of the Society for Neuroscience, 16(23), 7688–7698. 8922425 10.1523/JNEUROSCI.16-23-07688.1996 PMC6579073

[hbm25691-bib-0048] Roland, P. E. , Larsen, B. , Lassen, N. A. , & Skinhøj, E. (1980). Supplementary motor area and other cortical areas in organization of voluntary movements in man. Journal of Neurophysiology, 43(1), 118–136. 10.1152/jn.1980.43.1.118 7351547

[hbm25691-bib-0049] Rossignol, S. , Dubuc, R. , & Gossard, J.‐P. (2006). Dynamic sensorimotor interactions in locomotion. Physiological Reviews, 86(1), 89–154. 10.1152/physrev.00028.2005 16371596

[hbm25691-bib-0050] Schneider, W. , & Chein, J. M. (2003). Controlled & automatic processing: Behavior, theory, and biological mechanisms. Cognitive Science, 27(3), 525–559. 10.1207/s15516709cog2703_8

[hbm25691-bib-0051] Shibuya‐Tayoshi, S. , Sumitani, S. , Kikuchi, K. , Tanaka, T. , Tayoshi, S. , Ueno, S.‐I. , & Ohmori, T. (2007). Activation of the prefrontal cortex during the trail‐making test detected with multichannel near‐infrared spectroscopy. Psychiatry and Clinical Neurosciences, 61(6), 616–621. 10.1111/j.1440-1819.2007.01727.x 18081621

[hbm25691-bib-0052] Solodkin, A. , Hlustik, P. , Chen, E. E. , & Small, S. L. (2004). Fine modulation in network activation during motor execution and motor imagery. Cerebral Cortex (New York, N.Y.: 1991), 14(11), 1246–1255. 10.1093/cercor/bhh086 15166100

[hbm25691-bib-0053] Song, X.‐W. , Dong, Z.‐Y. , Long, X.‐Y. , Li, S.‐F. , Zuo, X.‐N. , Zhu, C.‐Z. , … Zang, Y.‐F. (2011). REST: A toolkit for resting‐state functional magnetic resonance imaging data processing. PLoS One, 6(9), e25031. 10.1371/journal.pone.0025031 21949842 PMC3176805

[hbm25691-bib-0054] Suzuki, M. , Miyai, I. , Ono, T. , Oda, I. , Konishi, I. , Kochiyama, T. , & Kubota, K. (2004). Prefrontal and premotor cortices are involved in adapting walking and running speed on the treadmill: An optical imaging study. NeuroImage, 23(3), 1020–1026. 10.1016/j.neuroimage.2004.07.002 15528102

[hbm25691-bib-0055] Swenor, B. K. , Muñoz, B. , & West, S. K. (2014). A longitudinal study of the association between visual impairment and mobility performance in older adults: The Salisbury eye evaluation study. American Journal of Epidemiology, 179(3), 313–322. 10.1093/aje/kwt257 24148711 PMC3954103

[hbm25691-bib-0056] Tannou, T. , Magnin, E. , Comte, A. , Aubry, R. , & Joubert, S. (2021). Neural activation in risky decision‐making tasks in healthy older adults: A meta‐analysis of fMRI data. Brain sciences, 11(8), 1043. 10.3390/brainsci11081043 34439662 PMC8393360

[hbm25691-bib-0057] Verghese, J. , Holtzer, R. , Lipton, R. B. , & Wang, C. (2009). Quantitative gait markers and incident fall risk in older adults. The Journals of Gerontology: Series A, Biological Sciences and Medical Sciences, 64(8), 896–901. 10.1093/gerona/glp033 PMC2709543 19349593

[hbm25691-bib-0058] Verghese, J. , Lipton, R. B. , Hall, C. B. , Kuslansky, G. , Katz, M. J. , & Buschke, H. (2002). Abnormality of gait as a predictor of non‐Alzheimer's dementia. The New England Journal of Medicine, 347(22), 1761–1768. 10.1056/NEJMoa020441 12456852

[hbm25691-bib-0059] Verghese, J. , Robbins, M. , Holtzer, R. , Zimmerman, M. , Wang, C. , Xue, X. , & Lipton, R. B. (2008). Gait dysfunction in mild cognitive impairment syndromes. Journal of the American Geriatrics Society, 56(7), 1244–1251. 10.1111/j.1532-5415.2008.01758.x 18482293 PMC2574944

[hbm25691-bib-0060] Verghese, J. , Wang, C. , Lipton, R. B. , Holtzer, R. , & Xue, X. (2007). Quantitative gait dysfunction and risk of cognitive decline and dementia. Journal of Neurology, Neurosurgery, and Psychiatry, 78(9), 929–935. 10.1136/jnnp.2006.106914 PMC1995159 17237140

[hbm25691-bib-0061] Wai, Y.‐Y. , Wang, J.‐J. , Weng, Y.‐H. , Lin, W.‐Y. , Ma, H.‐K. , Ng, S.‐H. , … Wang, C.‐H. (2012). Cortical involvement in a gait‐related imagery task: Comparison between Parkinson's disease and normal aging. Parkinsonism & Related Disorders, 18(5), 537–542. 10.1016/j.parkreldis.2012.02.004 22436654

[hbm25691-bib-0062] Ward, D. (2000). AlphaSim—Simultaneous inference for FMRI data. In AFNI. Milwaukee, WI: Medical College of Wisconsin. Retrieved from https://afni.nimh.nih.gov/pub/dist/doc/manual/AlphaSim.pdf

[hbm25691-bib-0063] Wilson, J. , Allcock, L. , Mc Ardle, R. , Taylor, J.‐P. , & Rochester, L. (2019). The neural correlates of discrete gait characteristics in ageing: A structured review. Neuroscience and Biobehavioral Reviews, 100, 344–369. 10.1016/j.neubiorev.2018.12.017 30552912 PMC6565843

[hbm25691-bib-0064] Woo, C.‐W. , Krishnan, A. , & Wager, T. D. (2014). Cluster‐extent based thresholding in fMRI analyses: Pitfalls and recommendations. NeuroImage, 91, 412–419. 10.1016/j.neuroimage.2013.12.058 24412399 PMC4214144

[hbm25691-bib-0065] Yang, J. F. , & Gorassini, M. (2006). Spinal and brain control of human walking: Implications for retraining of walking. The Neuroscientist: A Review Journal Bringing Neurobiology, Neurology and Psychiatry, 12(5), 379–389. 10.1177/1073858406292151 16957000

[hbm25691-bib-0066] Zwergal, A. , Linn, J. , Xiong, G. , Brandt, T. , Strupp, M. , & Jahn, K. (2012). Aging of human supraspinal locomotor and postural control in fMRI. Neurobiology of Aging, 33(6), 1073–1084. 10.1016/j.neurobiolaging.2010.09.022 21051105

