# Supplementary material for: Specific age‐correlated activation of top hierarchical motor control areas during gait‐like plantar stimulation: An fMRI study
Source: Hum Brain Mapp. 2021 Nov 5;43(2):833–43. doi: 10.1002/hbm.25691 (PMC8720193; doi:10.1002/hbm.25691)
Supplement: Supplementary file 1 — Appendix S1: Supporting information [file HBM-43-833-s001.pdf]

entire population (67 subjects) - CHAOTIC > REST

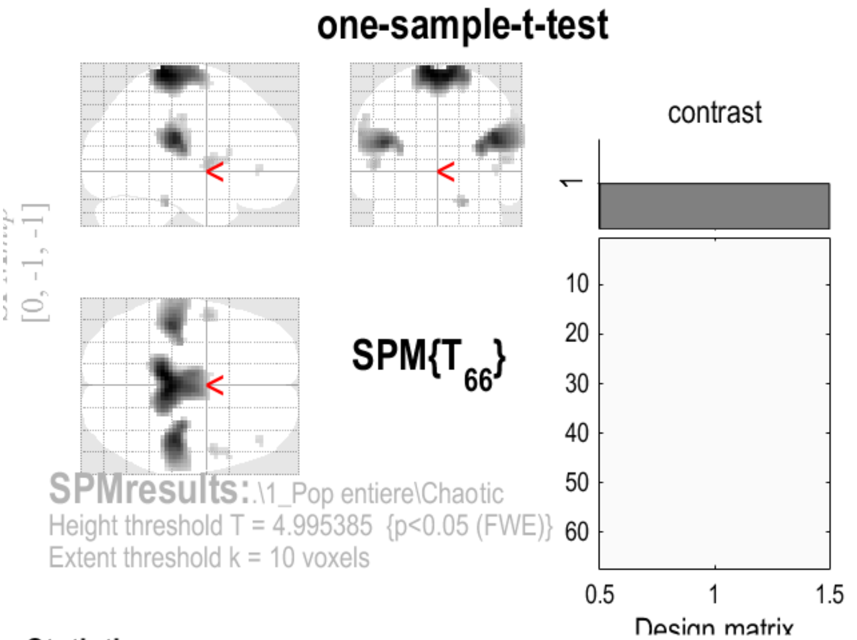

Statistics: ***p-values adjusted for search volume***

set-level p	set-level c	cluster-level $p_{FWE\text{-}corr}$	cluster-level $q_{FDR\text{-}corr}$	cluster-level k_E	cluster-level p_{uncorr}	peak-level $p_{FWE\text{-}corr}$	peak-level $q_{FDR\text{-}corr}$	peak-level T	peak-level $(Z_{\equiv})$	peak-level p_{uncorr}	mm	mm	mm
0.000	7	0.000		774	0.000	0.000	0.000	14.55	Inf	0.000	-6	-37	71
						0.000	0.000	14.21	Inf	0.000	3	-31	71
						0.000	0.000	10.13	7.83	0.000	-6	-16	74
		0.000		498	0.000	0.000	0.000	12.70	Inf	0.000	45	-28	20
						0.000	0.000	11.00	Inf	0.000	33	-22	17
		0.000		440	0.000	0.000	0.000	11.93	Inf	0.000	-45	-31	20
						0.003	0.000	5.87	5.25	0.000	-60	-22	38
		0.001		15	0.023	0.000	0.000	7.23	6.18	0.000	18	-34	-28
		0.000		41	0.002	0.000	0.000	6.80	5.90	0.000	-51	-1	5
		0.001		31	0.019	0.002	0.000	6.03	5.36	0.000	51	2	8
						0.017	0.000	5.34	4.85	0.000	54	14	8
		0.006		10	0.119	0.012	0.000	5.45	4.93	0.000	45	38	-1

table shows 3 local maxima more than 8.0mm apart

Height threshold: T = 5.00, p = 0.000 (0.050)
Extent threshold: k = 10 voxels, p = 0.036 (0.002)
Expected voxels per cluster, <k> = 2.198
Expected number of clusters, <c> = 0.00

Degrees of freedom = [1.0, 66.0]
FWHM = 13.0 12.9 12.7 mm mm mm; 4.3 4.3 4.2 {voxe
Volume: 1275885 = 47255 voxels = 526.9 resels
Voxel size: 3.0 3.0 3.0 mm mm mm; (resel = 78.95 voxe

[illegible]

entire population (67 subjects) - ORGANIZED > REST

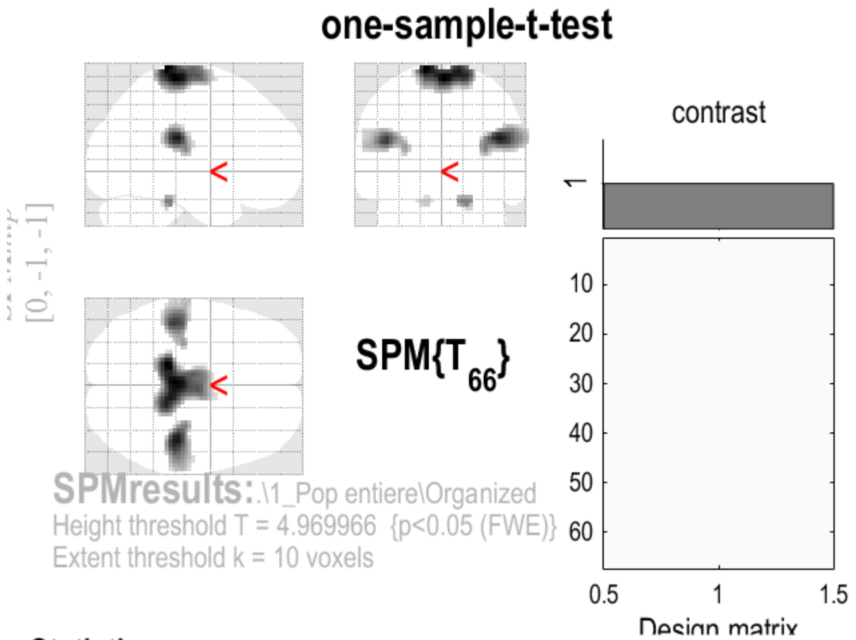

Statistics: ***p-values adjusted for search volume***

set-level		cluster-level				peak-level					mm mm mm		
p	c	$p_{FWE-corr}$	$q_{FDR-corr}$	k_E	p_{uncorr}	$p_{FWE-corr}$	$q_{FDR-corr}$	T	$(Z_{\equiv})$	p_{uncorr}			
0.000	5	0.000		689	0.000	0.000	0.000	13.52	Inf	0.000	-15	-37	77
						0.000	0.000	13.23	Inf	0.000	0	-31	68
						0.000	0.000	12.39	Inf	0.000	12	-34	74
		0.000		372	0.000	0.000	0.000	12.38	Inf	0.000	45	-28	23
						0.000	0.000	8.64	7.04	0.000	33	-22	17
		0.000		297	0.000	0.000	0.000	11.03	Inf	0.000	-45	-31	20
						0.000	0.000	7.56	6.40	0.000	-30	-25	17
		0.000		30	0.002	0.000	0.000	8.62	7.03	0.000	18	-34	-25
		0.004		13	0.076	0.001	0.000	6.14	5.44	0.000	-15	-37	-25

table shows 3 local maxima more than 8.0mm apart

Height threshold: T = 4.97, p = 0.000 (0.050) Degrees of freedom = [1.0, 66.0]
Extent threshold: k = 10 voxels, p = 0.045 (0.002) FWHM = 13.5 13.3 13.0 mm mm mm; 4.5 4.4 4.3 {voxe
Expected voxels per cluster, <k> = 2.432 Volume: 1275885 = 47255 voxels = 483.7 resels
Expected number of clusters, <c> = 0.00 Voxel size: 3.0 3.0 3.0 mm mm mm; (resel = 86.01 voxe
[illegible]

18-38 years (20 subjects) - CHAOTIC > REST

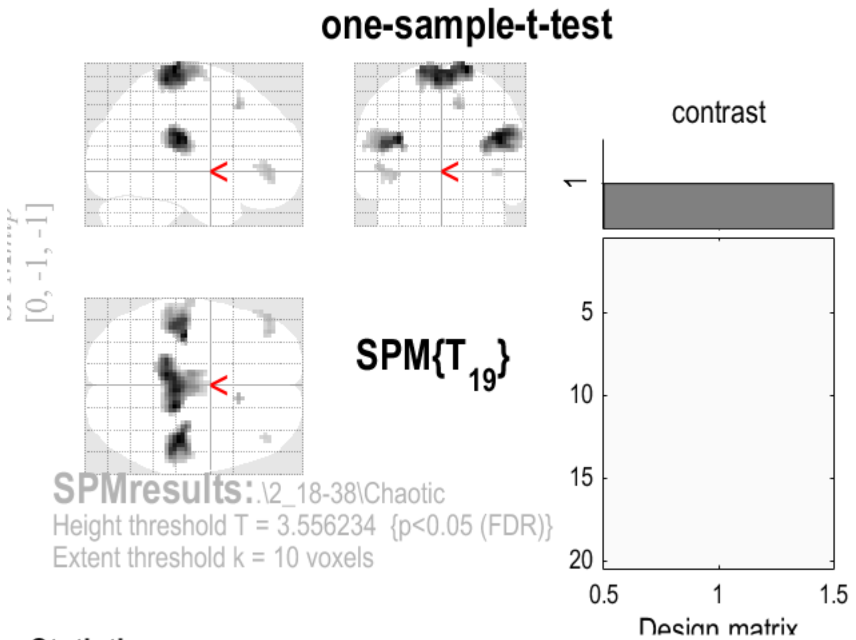

Statistics: ***p-values adjusted for search volume***

set-level		cluster-level				peak-level					mm mm mm		
p	c	$p_{FWE-corr}$	$q_{FDR-corr}$	k_E	p_{uncorr}	$p_{FWE-corr}$	$q_{FDR-corr}$	T	$(Z_{\equiv})$	p_{uncorr}			
0.016	6	0.000		486	0.000	0.005	0.001	8.00	5.23	0.000	18	-31	77
						0.009	0.001	7.66	5.11	0.000	-6	-37	68
						0.012	0.001	7.47	5.04	0.000	6	-31	71
		0.000		299	0.000	0.005	0.001	7.90	5.20	0.000	45	-28	23
						0.111	0.001	6.19	4.53	0.000	33	-22	17
		0.000		258	0.000	0.007	0.001	7.75	5.14	0.000	-36	-22	23
						0.049	0.001	6.71	4.75	0.000	-45	-31	17
		0.755		15	0.164	0.791	0.009	4.63	3.74	0.000	12	20	47
						0.999	0.039	3.72	3.18	0.001	9	20	59
		0.393		47	0.058	0.897	0.013	4.40	3.61	0.000	-48	44	-1[illegible]
						0.932	0.016	4.30	3.55	0.000	-39	44	-[illegible]
						0.994	0.029	3.89	3.29	0.000	-54	32	[illegible]

table shows 3 local maxima more than 8.0mm apart

Height threshold: T = 3.56, p = 0.001 (1.000)
Extent threshold: k = 10 voxels, p = 0.234 (0.864)
Expected voxels per cluster, <k> = 7.587
Expected number of clusters, <c> = 2.00

Degrees of freedom = [1.0, 19.0]
FWHM = 13.9 13.7 13.3 mm mm mm; 4.6 4.6 4.4 {voxe[illegible]
Volume: 1461456 = 54128 voxels = 514.3 resels
Voxel size: 3.0 3.0 3.0 mm mm mm; (resel = 9[illegible]

[illegible]

18-38 years (20 subjects) - ORGANIZED > REST

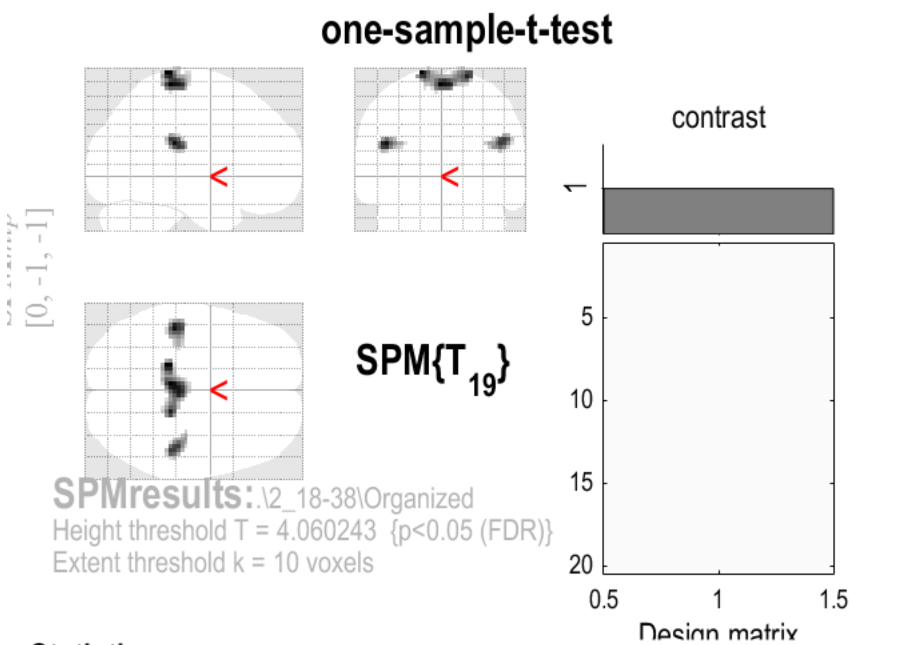

Statistics: ***p-values adjusted for search volume***

set-level		cluster-level				peak-level					mm mm mm		
p	c	$p_{FWE-corr}$	$q_{FDR-corr}$	k_E	p_{uncorr}	$p_{FWE-corr}$	$q_{FDR-corr}$	T	$(Z_{\equiv})$	p_{uncorr}			
0.024	3	0.001		228	0.000	0.012	0.003	7.47	5.04	0.000	-3	-34	68
						0.014	0.003	7.40	5.02	0.000	-18	-37	77
						0.032	0.003	6.91	4.83	0.000	18	-34	74
		0.008		65	0.002	0.023	0.003	7.13	4.92	0.000	-45	-31	23
		0.017		68	0.005	0.061	0.004	6.51	4.66	0.000	48	-34	26

table shows 3 local maxima more than 8.0mm apart

Height threshold: T = 4.06, p = 0.000 (0.970)
Extent threshold: k = 10 voxels, p = 0.172 (0.454)
Expected voxels per cluster, <k> = 5.694
Expected number of clusters, <c> = 0.61
[illegible]

Degrees of freedom = [1.0, 19.0]
FWHM = 14.6 14.1 13.8 mm mm mm; 4.9 4.7 4.6 {voxe
Volume: 1461456 = 54128 voxels = 456.9 resels
Voxel size: 3.0 3.0 3.0 mm mm mm; (resel = 105.45 vox

38-58 years (23 subjects) - CHAOTIC > REST

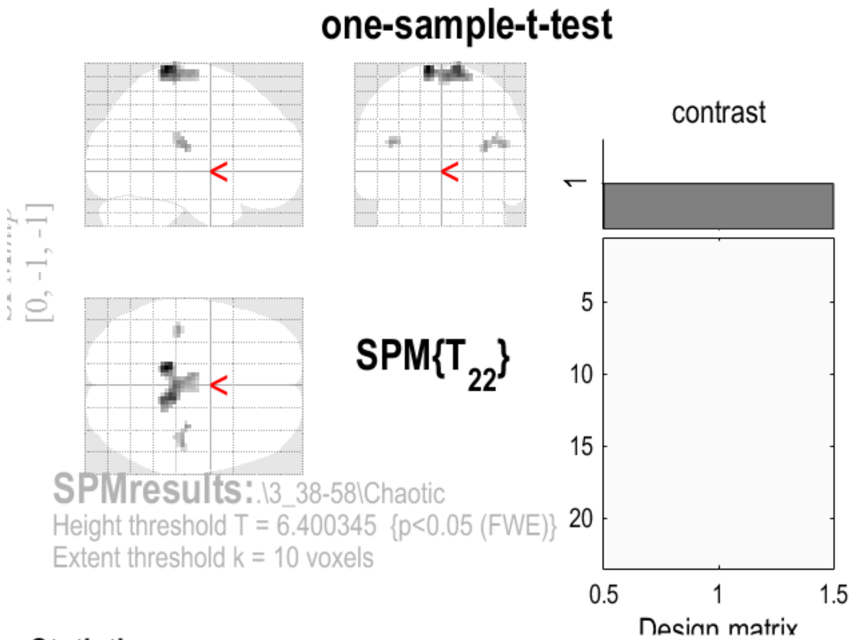

Statistics: ***p-values adjusted for search volume***

set-level		cluster-level				peak-level					mm mm mm		
p	c	$p_{FWE-corr}$	$q_{FDR-corr}$	k_E	p_{uncorr}	$p_{FWE-corr}$	$q_{FDR-corr}$	T	$(Z_{\equiv})$	p_{uncorr}			
**0.000**	**3**	**0.000**		**202**	**0.000**	**0.000**	**0.000**	**10.05**	**6.09**	**0.000**	**-12**	**-37**	**77**
						0.000	0.000	8.91	5.74	0.000	12	-34	74
						0.001	0.000	8.40	5.57	0.000	3	-31	71
		**0.000**		**31**	**0.000**	**0.003**	**0.000**	**7.65**	**5.29**	**0.000**	**33**	**-22**	**17**
						0.004	0.000	7.49	5.23	0.000	48	-25	17
						0.020	0.000	6.81	4.95	0.000	42	-28	26
		**0.000**		**13**	**0.001**	**0.004**	**0.000**	**7.51**	**5.23**	**0.000**	**-42**	**-28**	**20**

table shows 3 local maxima more than 8.0mm apart

Height threshold: T = 6.40, p = 0.000 (0.050)
Extent threshold: k = 10 voxels, p = 0.002 (0.00
Expected voxels per cluster, <k> = 0.894
Expected number of clusters, <c> = 0.00
[illegible]

Degrees of freedom = [1.0, 22.0]
FWHM = 12.3 12.2 11.9 mm mm mm; 4.1 4.1 4.0 {voxe
Volume: 1396008 = 51704 voxels = 694.0 resels
Voxel size: 3.0 3.0 3.0 mm mm mm; (resel = 66.07 voxe

38-58 years (23 subjects) - ORGANIZED > REST

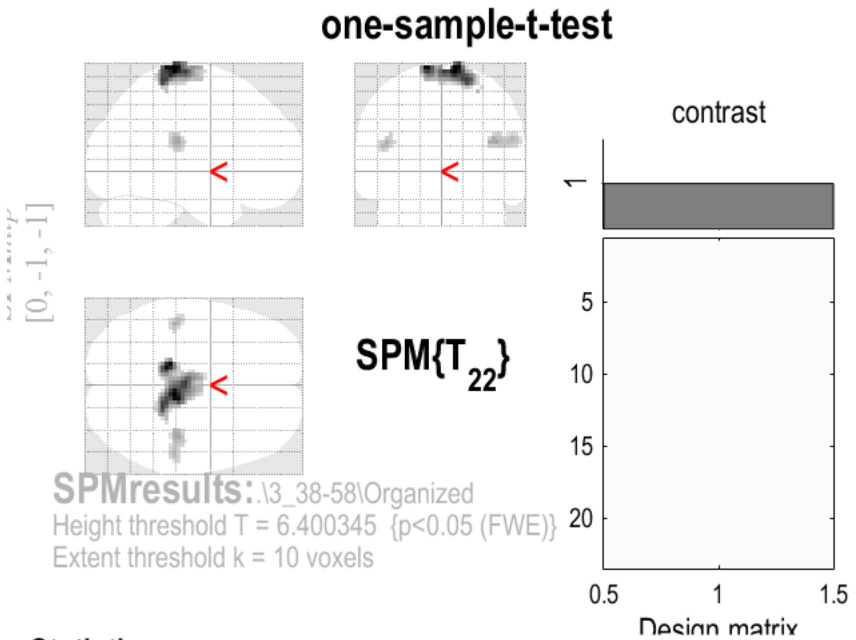

Statistics: ***p-values adjusted for search volume***

set-level		cluster-level				peak-level					mm mm mm		
p	c	$p_{FWE-corr}$	$q_{FDR-corr}$	k_E	p_{uncorr}	$p_{FWE-corr}$	$q_{FDR-corr}$	T	($Z_{\equiv}$)	p_{uncorr}			
0.000	3	0.000		395	0.000	0.000	0.000	13.53	6.94	0.000	9	-31	74
						0.000	0.000	12.95	6.82	0.000	-15	-34	77
						0.000	0.000	11.50	6.48	0.000	0	-22	74
		0.000		75	0.000	0.001	0.000	8.15	5.48	0.000	42	-28	20
						0.002	0.000	7.77	5.33	0.000	54	-31	23
		0.000		32	0.000	0.002	0.000	7.91	5.39	0.000	-45	-31	20

table shows 3 local maxima more than 8.0mm apart

Height threshold: T = 6.40, p = 0.000 (0.050)
Extent threshold: k = 10 voxels, p = 0.003 (0.000)
Expected voxels per cluster, <k> = 0.933
Expected number of clusters, <c> = 0.00
[illegible]

Degrees of freedom = [1.0, 22.0]
FWHM = 12.5 12.3 12.0 mm mm mm; 4.2 4.1 4.0 {voxe
Volume: 1396008 = 51704 voxels = 665.3 resels
Voxel size: 3.0 3.0 3.0 mm mm mm; (resel = 68.92 voxe

58-78 years (24 subjects) - CHAOTIC > REST

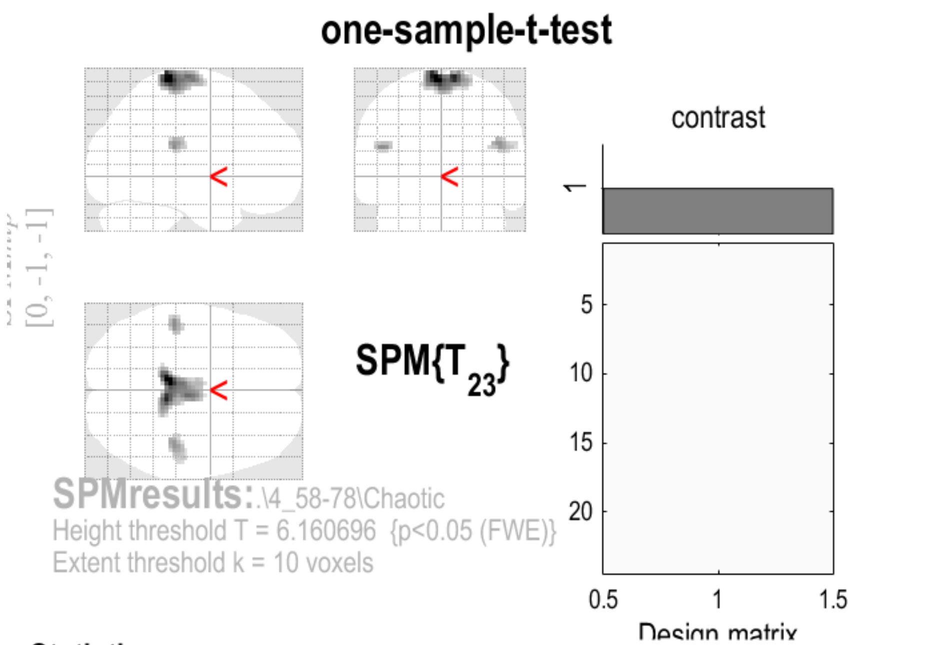

Statistics: ***p-values adjusted for search volume***

set-level		cluster-level				peak-level					mm mm mm		
p	c	$p_{FWE-corr}$	$q_{FDR-corr}$	k_E	p_{uncorr}	$p_{FWE-corr}$	$q_{FDR-corr}$	T	$(Z_{\equiv})$	p_{uncorr}			
0.000	3	0.000		337	0.000	0.000	0.000	12.12	6.72	0.000	-6	-37	71
						0.000	0.000	11.04	6.45	0.000	9	-37	74
						0.000	0.000	9.15	5.89	0.000	6	-19	74
		0.000		35	0.001	0.001	0.000	8.30	5.59	0.000	-48	-31	20
		0.000		59	0.000	0.001	0.000	8.09	5.51	0.000	45	-31	23

table shows 3 local maxima more than 8.0mm apart

Height threshold: T = 6.16, p = 0.000 (0.050)
Extent threshold: k = 10 voxels, p = 0.009 (0.000)
Expected voxels per cluster, <k> = 1.319
Expected number of clusters, <c> = 0.00
[illegible]

Degrees of freedom = [1.0, 23.0]
FWHM = 13.4 13.4 13.0 mm mm mm; 4.5 4.5 4.3 {voxe
Volume: 1297539 = 48057 voxels = 486.6 resels
Voxel size: 3.0 3.0 3.0 mm mm mm; (resel = 86.97 voxe

58-78 years (24 subjects) - ORGANIZED > REST

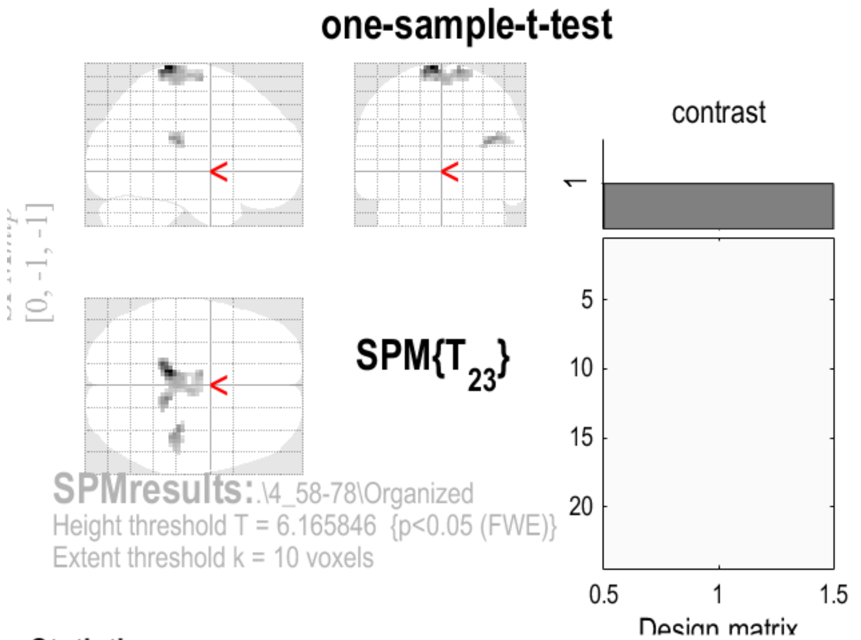

Statistics: ***p-values adjusted for search volume***

set-level		cluster-level				peak-level					mm mm mm		
p	c	$p_{FWE-corr}$	$q_{FDR-corr}$	k_E	p_{uncorr}	$p_{FWE-corr}$	$q_{FDR-corr}$	T	$(Z_{\equiv})$	p_{uncorr}			
0.000	2	0.000		185	0.000	0.000	0.000	11.37	6.53	0.000	-9	-34	77
						0.000	0.000	9.44	5.98	0.000	-15	-40	74
						0.001	0.000	8.34	5.60	0.000	12	-37	74
		0.000		49	0.000	0.001	0.000	8.04	5.49	0.000	36	-28	20

table shows 3 local maxima more than 8.0mm apart

Height threshold: T = 6.17, p = 0.000 (0.050)
Extent threshold: k = 10 voxels, p = 0.009 (0.000)
Expected voxels per cluster, <k> = 1.302
Expected number of clusters, <c> = 0.00
[illegible]

Degrees of freedom = [1.0, 23.0]
FWHM = 13.4 13.3 13.0 mm mm mm; 4.5 4.4 4.3 {voxe
Volume: 1297539 = 48057 voxels = 491.6 resels
Voxel size: 3.0 3.0 3.0 mm mm mm; (resel = 86.08 voxe
